# Risk factors of postoperative airway obstruction complications in children with oral floor mass

**DOI:** 10.1515/med-2024-0959

**Published:** 2024-07-02

**Authors:** Ying Liu, Chen Zhuo, Yujiao Guo, Yang Jiang, Mingzhe Li, Yangyang Zhao, Xiaolu Wu, Guoxia Yu

**Affiliations:** Department of Stomatology, National Clinical Research Center for Respiratory Diseases, Beijing Children’s Hospital, Capital Medical University National Center for Children’s Health, Beijing 100045, China

**Keywords:** floor-of-mouth mass, airway obstruction, risk factors, surgical treatment, children

## Abstract

The aim of the present study was to explore the risk factors of postoperative airway complications in children with oral floor mass. The first choice of auxiliary examination method for children with oral floor mass is also proposed. This retrospective study included 50 children with floor-of-mouth (FOM) masses. Medical records were reviewed, and information on age of onset, functional impacts present, age at consultation, imaging findings, history of preoperative aspiration, pathology findings, properties of biopsied fluid, treatment modality, postoperative outcomes, and operation were recorded. A total of 20 patients exhibited functional impacts such as difficulty in breathing and feeding. Ultrasound examination was performed in 28 cases; and magnetic resonance imaging, in 38 cases. The diagnosis was lymphatic malformation in 12 cases, developmental cyst in 29 cases, and solid mass in 7 cases. There were 28 cases of surgical resection, 9 cases underwent multiple puncture volume reduction followed by surgery, 11 cases treated using sclerotherapy injection, and 1 case treated using sclerotherapy injection and surgical resection. Young age, functional impact, and high grade of lymphatic duct malformation increased the risk of surgical treatment. B-scan ultrasound is the first choice for the diagnosis of FOM masses in children.

## 1 Introduction

The oral cavity is the starting point of the gastrointestinal and respiratory tracts and has complex anatomical and functional structures. Floor-of-mouth (FOM) masses have complex and varied functional impacts. They may elevate the body of the tongue, which may affect feeding, swallowing, articulation, and push the root of the tongue backward, which may cause breathing difficulty and cause asphyxia in serious cases [1]. FOM masses in younger children, particularly newborns, may interfere with breathing and feeding. Owing to specific characteristics of children, the FOM has a loose tissue structure, and it is more susceptible to life-threatening conditions such as bleeding and infection. Therefore, clinical management is more difficult than that in adults, and there are several possible diagnoses and different treatment principles for children with FOM masses [2,3,4]. Accurate assessment of FOM masses and treatment with the right approach and right time are essential

We reviewed 50 cases of children with FOM masses and proposed factors related to airway obstruction risk after surgery for FOM masses in children.

## 2 Materials and methods

### 2.1 Study participants

Children with FOM masses who were hospitalized for surgical treatment between June 2009 and December 2021 at the Department of Oral and Maxillofacial Surgery, Beijing Children’s Hospital, Capital Medical University, were selected.

The inclusion criteria were as follows: (1) preliminary diagnosis of FOM mass in medical history, clinical examination, and imaging and (2) treatment by surgical removal of mass or drug injection after admission.

The exclusion criteria were as follows: (1) diagnosed with ranula or mucous cyst on clinical examination and aspiration biopsy, imaging, or pathological examination and (2) having with FOM mass but failed to undergo treatment because of systemic diseases or other reasons.

### 2.2 General information

The following data were collected and analyzed: age of onset, age at consultation, and whether there were any effects on breathing and feeding.

### 2.3 Imaging findings

We identified whether the patients underwent ultrasound examination or magnetic resonance imaging (MRI) and analyzed the findings thereof: single cyst, multiple cysts, solid cyst, and uncertain for ultrasound examination/MRI. The postoperative airway risk was then predicted, and the accuracy of the prediction was calculated.

### 2.4 Biopsy

We determined whether preoperative aspiration biopsy was performed and the aspiration fluid properties (clear, off-white, other colors, could not be biopsied) and whether multiple preoperative cyst punctures for fluid volume reduction or fine needle aspiration (FNA) cytology was performed.

### 2.5 Treatment modality and postoperative complications

The following data were collected and analyzed: surgical resection, multiple volume reductions and surgical resection, sclerotherapy injection, sclerotherapy injection, surgical resection, whether there was postoperative pediatric intensive care unit (PICU) admission, and whether recurrence occurred.

### 2.6 Diagnostic data

Statistical data on preliminary diagnosis, pathological diagnosis, and diagnosis at discharge were collected. Diagnostic data were classified into one of the three categories: solid mass, lymphatic malformation, and developmental cyst. The agreement between B-scan ultrasound examination and properties of the mass and between B-scan ultrasound and aspiration and diagnosis were determined.

### 2.7 Statistical analysis

SPSS23.0 was used to analyze the correlation between the age of onset and age at surgery to their effect on breathing, eating, puncture volume reduction, diagnostic information, treatment methods, and postoperative PICU admission. We used the Pearson chi square test (Pearson *X* ^2^) method, and the *P*-value was set to 0.05. If *P* < 0.05, the significance level was reached, indicating that this factor has a significant difference in postoperative PICU. On this basis, calculate the Cramer’s *V*, with a significance *P* value of 0.05. If *P* < 0.05, it indicates a significant correlation between this factor and postoperative PICU.


**Ethical approval:** This study was conducted following the principles of the Declaration of Helsinki (revised by the World Medical Association in 2013) and approved by the Ethics Committee of the Beijing Children’s Hospital, Capital Medical University (approval number, [2022]-E-109-R).
**Informed consent:** As this study was retrospective and did not contain identifying patient information, the need for informed consent was waived.

## 3 Results

After screening by applying the inclusion and exclusion criteria, a total of 50 children, 32 boys, and 18 girls aged between 2 months and 13 years were included in the study. Details of the age of onset and treatment of the patients are shown in Table 4. Of the 41 children with an age of onset <2 years, 28 were detected at birth, accounting for 56% of the total number of cases. The median age at the time of treatment was 16.5 months.

Of the 33 children who underwent puncture aspiration of cystic fluid, 12 had clear or slightly bloody fluid, which was considered a lymphatic malformation, and 16 had yellow, creamy white, or brown fluid, which was considered a developmental cyst. There were two cases of FNA cytology, one of primitive neuroectodermal tumor and one of alveolar soft part sarcoma (Table 1).

**Table 1:** Functional impacts, imaging findings, aspiration biopsy results, diagnosis, and treatment data of the 50 cases of FOM masses in children

		Cases	Percentage (%)
**Functional impact**		20	40.0
	Respiration	12	24.0
	Feeding	13	26.0
**B-scan ultrasound**		28	56.0
	Single	16	32.0
	Multiple	11	22.0
	Solid (nodular)	1	2.0
**MRI**		38	76.0
	Single	20	40.0
	Multiple	14	28.0
	Solid (nodular)	4	8.0
**Aspiration data**		33	66.0
	Clear fluid	12	24.0
	Other colors	16	32.0
	Not possible	3	6.0
	FNAC	2	4.0
	Multiple cystic reduction	9	18.0
**Treatment modality**			
	Surgical resection	28	56.0
	Reduction + surgery	9	18.0
	Sclerotherapy injection	11	22.0
	Sclerotherapy + surgery	1	2.0
**Diagnostic data**			
	Lymphatic	12	24.0
	Malformation developmental cyst	29	58.0
	Solid (nodular) masses	7	14.0

FNAC, fine needle aspiration cytology; FOM, floor of mouth; MRI, magnetic resonance imaging.

The agreement between B-scan ultrasound results and properties of the mass and between B-scan ultrasound and aspiration biopsy examination and diagnosis is shown in Table 2.

**Table 2:** Agreement between B-scan ultrasound and mass properties and between B-scan ultrasound + aspiration biopsy and diagnosis

	Cases	Accurately diagnosed cases	Agreement rate (%)
**B-scan ultrasound**	28		
Lymphatic malformation	10	10	100
Developmental cyst	14	13	92.6
Solid (nodular) mass	2	1	50
**B-scan ultrasound + aspiration biopsy**	18		
Lymphatic malformation	10	10	100
Developmental cyst	8	8	100

The results of the correlation analysis between the age of onset and age at surgery to their effect on breathing, eating, puncture volume reduction, diagnostic information, treatment methods, and PICU admission after surgery are shown in Table 3. The results show statistically significant (*P* value 0.010) difference in feeding on PICU after surgery, with a correlation coefficient of 0.365 (Cramer’s *V* = 0.365, *P* = 0.01).

**Table 3:** Analysis of related factors of postoperative PICU admission

	Postoperative PICU admission	
	Pearson *X* ^2^	Significance (bilateral)
Age at onset	3.178	0.204
Age at treatment	7.415	0.116
Respiration	3.429	0.064
Feeding**	6.652*	0.010
Multiple cystic reduction	0.374	0.541
Diagnostic data	3.464	0.177
Treatment modality	7.857	0.097

*Significant differences at 0.05 level (bilateral).

**Cramer’s *V* is 0.365. Significance is 0.01. Significant correlation at 0.05 level.

PICU, pediatric intensive care unit.

Seven children were admitted to the PICU after puncture or surgical treatment (Table 4). In five cases (71.4%), the children were younger than 1 year. There were three cases (42.9%) of lymphatic malformation.

**Table 4:** Age of the 50 patients with FOM mass and of those admitted to the PICU

	Cases of age group				Total
	<1 year	1–2 years	2–4 years	>4 years	
Age at onset	36	5	0	9	50
Age at treatment	21	11	7	11	50
Postoperative PICU admission	5	1	0	1	7

FOM, floor of mouth; PICU, pediatric intensive care unit.

## 4 Discussion

If children are required to admit to PICU, it will increase the cost of treatment, and increase the economic burden on their families. The purpose of this study was to identify the relevant factors that increase the risk of airway obstruction after treatment of oral floor masses and explore how to avoid them.

### 4.1 High-risk factors for surgical complications

The results of this study showed that there was a significant correlation between impaired feeding before the operation of FOM mass and admission to the PICU after the operation. This may be due to a mass large in size and located posteriorly at the bottom of the mouth, causing difficulties in eating.

Although correlation analysis showed that there was no significant correlation between the age of surgery, diagnosis information, and entering PICU after surgery, from Table 4, we can see that of the seven children who entered PICU after operation, five were younger than 1 year old. Young age is a risk factor for serious postoperative airway obstruction. Ueno et al. reported the airway status of 518 children with head and neck lymphatic malformations after treatment, of whom 43 required tracheotomy and 32 were younger than 1 year [5]. Therefore, we suggest that “active observation” should be practiced for children with FOM masses as well as children with lymphatic malformation; children without functional impairment can be closely observed and treated at a later age.

In the present study, three of the seven children admitted to the PICU had lymphatic malformations. In 1995, Serres et al. graded lymphatic malformations in the head and neck based on anatomical sites to predict preoperative and postoperative complications [6]. In 2001, Hamoir et al. further validated this staging method, classifying head and neck lymphatic malformations into six stages according to anatomical site: (I) unilateral infrahyoid; (II) unilateral suprahyoid; (III) unilateral suprahyoid and infrahyoid; (IV) bilateral suprahyoid; (V) bilateral suprahyoid and infrahyoid; and (VI) bilateral infrahyoid. Higher grades correspond to higher risk of preoperative and postoperative complications, including infection, bleeding, airway compression, and feeding difficulties [7]. Therefore, lymphatic malformations in the FOM should be evaluated before treatment to assess the risk of postoperative complications, which can be predicted using the Serres classification system. Balakrishnan et al. also concluded that treatment modality is not associated with postoperative complications, and the higher the stage, the higher the risk of postoperative admission to the ICU and tracheotomy [8].

Due to the above-mentioned reasons, regular aspiration can be used as a palliative treatment when the FOM mass causes breathing and feeding difficulties in children for whom surgery is not appropriate, to delay surgery to a later age and reduce airway complication risk. It should not be performed too early as younger children have a smaller space in the FOM, resulting in a higher risk of respiratory and feeding difficulties after aspiration.

### 4.2 Selection and application of imaging methods

Consistent with the Schwanke study, the present study showed that B-scan ultrasound was highly accurate for determining the properties of the FOM mass: single or multiple cysts or solid (nodular). In addition, B-scan ultrasound is noninvasive and radiation-free and can serve as the preferred auxiliary examination method for FOM masses.

MRI helps visualize the extent of the FOM mass and accurately demonstrate the relationship between the mass and surrounding anatomical structures. Lymphatic malformations have characteristic manifestations on MRI: lobulated, separated masses with long T1 and T2 signals; microcystic lymphatic malformations have no significant enhancement; and macrocystic lymphatic malformations show only marginal and interval enhancement. The present study also suggests that MRI can predict postoperative airway obstruction risk more accurately prior to surgery.

Therefore, we believe that further MRI can be performed in the following cases: (1) B-scan ultrasound results suggest a solid mass and a multi-cystic lesion that cannot be clearly identified as a lymphatic malformation, (2) prior to drug injection or surgical treatment, and (3) contents have not been punctured.

### 4.3 FOM mass treatment principles

In this study, “observation” was used as a treatment option for children with FOM masses. The choice between observation and invasive treatment depended on whether the child had difficulty in breathing, feeding, and articulating or other functional impairments. The results were consistent with those of Bonilla-Velez et al. [9] who in 2021 proposed that children with lymphatic duct malformations should be treated with “active observation” because invasive treatment increases the number of treatments for children under 6 months of age and lymphatic duct malformations tend to resolve spontaneously [10]. In other words, children with lymphatic malformations without any functional impairment can be observed and only treated if any impairment of breathing or feeding develops [9].

In the present study, this principle was followed in the treatment of children with FOM masses, so that although 36 children developed the disease at under 1 year of age, only 21 were treated, which reduced postoperative airway obstruction risk.

The limitation of this study is that due to the small sample size of children under 1 year old, it is not possible to further group children under 1 year old. Further research with larger samples is needed.
